# Relationships between training load and wellbeing measures across a full season: a study of Turkish national youth wrestlers

**DOI:** 10.5114/biolsport.2023.116009

**Published:** 2022-06-01

**Authors:** Hadi Nobari, Georgian Badicu, Zeki Akyildiz, Filipe Manuel Clemente

**Affiliations:** 1 Faculty of Sport Sciences, University of Extremadura, 10003 Cáceres, Spain; 2 Transilvania University of Brasov, Brasov, Romania; 3 Movement and Training Science Department, Gazi University, Ankara, Turkey; 4 Escola Superior Desporto e Lazer, Instituto Politécnico de Viana do Castelo, Rua Escola Industrial e Comercial de Nun’Álvares, 4900-347 Viana do Castelo, Portugal; 5 Instituto de Telecomunicações, Delegação da Covilhã, Lisboa 1049-001, Portugal; 6 Research Center in Sports Performance, Recreation, Innovation and Technology (SPRINT), 4960-320 Melgaço, Portugal

**Keywords:** Hooper index, Recovery, Workload monitoring, Performance, ACWR

## Abstract

The two aims of this study were: (i) to analyse the correlations between weekly acute workload (wAW), chronic workload, acute/chronic workload rate (wACWR), training monotony and strain and the weekly (w) reports of delayed onset muscle soreness, wFatigue, wStress, wSleep quality, and the Hooper indicator (wHI); (ii) to analyse the relationships between early, mid and end preparation season (PS) and the full PS. Ten elite young wrestlers participated in this study. The subjects who were included in this research were wrestlers participating in competitions organized by the National Turkish Wrestling Federation. The subjects were monitored for 32 weeks and were divided into three time periods: early PS, W1 to W11; mid PS, W12 to W22; and end PS, W23 to W32. Very large correlations were found for wAW and wACWR with wFatigue and wHI during end PS. Also, the same workload parameters were significantly correlated with wStress (r = 0.66; P = 0.03), wSleep (r = 0.78; P = 0.004), and wHI (r = 0.72; P = 0.01) during mid PS. The results of this study offer new perspectives for specialists regarding the perceived level of load and the variations of wellbeing during a PS at the level of elite young wrestlers.

## INTRODUCTION

In elite sport practice and in research, the analysis of internal and external training loads has become a critical issue. In this regard, monitoring the athletes’ internal training loads is important for understanding whether athletes are positively adapting to their training programme. Bourdon et al. (2017) stated that the measures of training load can be categorized as either internal or external, where external training loads are objective measures of the work performed by the athlete (speed, volume, acceleration, etc.) [1]. Internal training load is defined as the relative physiological and psychological stressors imposed on the athlete during training or competition. The same authors stated that there exist various methods for measuring internal load, such as rating of perceived exertion (RPE), session RPE (sRPE), training impulse (TRIMP), heart rate indices, blood lactate, oxygen uptake and/or psychological scales and questionnaires [1–3]. At present, especially the sRPE [2, 3] and the acute:chronic workload ratio (ACWR) [4] methods are being discussed, whereas sRPE has been extensively investigated and seems to be a valid tool for measuring internal training load in a variety of sports [3], especially in swimming [5].

Complementary, subjective daily wellbeing questionnaires have become increasingly prominent as a quick and easy method of understanding an athlete’s readiness to train and can incorporate questions surrounding an athlete’s sleep, stress levels, fatigue, etc. [6]. There is a large body of studies demonstrating the change in wellbeing questionnaires over the course of a pre- or full-season period [7]. Fullagar et al. [9] found that the perceptions of wellbeing variables (i.e., sleep quality, delay onset muscle soreness [DOMS], energy, and overall wellness) fell to the lowest level the day after a rugby league or American football match, but did not recover to baseline levels for at least four days after the match [7]. Furthermore, research has shown that a drop in perceptions of wellbeing can lead to reductions in external training load output in elite adult soccer and Australian rules players [8]. Additional, this study failed to quantify the association between training load and wellbeing in adolescent athletes [9].

In a study performed by Rossi et al. [10] on 22 elite soccer players, in 160 training sessions and 35 matches during the 2015/2016 season, it was found that the training workloads performed in the previous week had a strong effect on perceived exertion and training load. On the other hand, the analysis of this prediction showed higher accuracy for medium RPE and S-RPE values compared with the extremes.

Additionally, the relationship between players’ wellness profile and training load has received growing interest in recent years. The literature provides significant interactions between DOMS, stress, fatigue perception, and sleep quality [11].

However, regarding our study, there is no recent research analysing the relationship between the training load and wellbeing of youth wrestlers, which justifies this research. We found only a study conducted in Turkey, where they measured the level of anxiety in 50 wrestlers [11], or the somatotype and anthropometric assessment of wrestlers. A similar study by Lupo et al. [12] on five female and four male taekwondo athletes did not reveal any difference for gender. The internal training loads of competitive sessions (Edwards: 228 ± 40 arbitrary units, AU) were higher than those of pre-competitive sessions (192 ± 26 AU; P = 0.04). Although all data collections achieved significant correlations between Edwards’ and session-RPE methods, a strong relationship (r = 0.71, P < 0.001) emerged only for PC sessions evaluated at 30 minutes of the recovery phase.

Based on the above observations, this study aimed: (1) to analyse the correlations between weekly acute workload (wAW), weekly chronic workload (wCW), wACWR, weekly training monotony (wTM), weekly training strain (wTS) and the weekly reports of delayed onset muscle soreness (DOMS), fatigue, stress, sleep quality, and the Hooper indicator (HI); (2) to analysis the relationships of early, mid and end preparation season (PS) with the full PS.

## MATERIALS AND METHODS

### Participants

Participants were ten elite juvenile wrestlers (mean ± standard deviation (SD); age, 16 ± 0.7 years; height, 163 ± 4.8 cm; body mass, 57.7 ± 9.0 kg; VO_2max_, 48.7 ± 1.4 ml · kg^−1^ · min^−1^), who were preparing to participate in competitions in the National Turkish Wrestling Federation. Inclusion criteria for the youth wrestlers recruited for this study were: (i) to have taken part in ≥ 90% of the training sessions of the whole PS; (ii) to not participate in separate exercises throughout the PS; (iii) to attend the national team camp during the PS. Hence, the type of feeding, rest, sleep, and accommodation temperature were the same for all wrestlers during the PS. PS training days included the following: (i) wrestling-specific technical and skills with moderate-intensity aerobic training; (ii) wrestling-specific technical and skills with anaerobic training; (iii) hypertrophy training for muscular neuromuscular adaptation; (iv) containing special moves in wrestling tactical and full body-weight training, plyometric training; (v) rest day; (vi) velocity and power training; (vii) and finally, competitive wrestling within the team. These wrestlers had at least 6 years of training experience. Before the start of the study, all wrestlers were enlisted, and with their parents were notified of the study’s stages, and consent was signed by all of them. This study was approved by the Ethics Committee of the Afyon Kocatepe University. The entire study follows the Helsinki Declaration regarding human experimentation.

### Sample size

Previous studies have reported large to very large correlations of workload variables with wellbeing in youth athletic and individual sports [13–16]. To obtain the sample size, the results were analysed with a minimum power of 0.80. Consequently, assuming the distribution to be two-tailed, with an α error of less than 0.05 and a large size effect, 10 wrestlers are required to reach 83.9% actual power to correlate workload variables with wellbeing.

### Study design

The present study is a longitudinal descriptive study on under-17-year-old elite wrestlers of the Turkish national team in the 2017–2018 season. Daily information was collected in each training session for 32 weeks during the full PS. The whole monitoring was divided into three periods: early PS, W1 to W11; mid PS, W12 to W22; and end PS, W23 to W32 (Table 1). Throughout the PS, the minimum number of exercises recorded per week was three sessions. All wrestlers had three years of self-reported (RPE and HI) history of the Hooper Questionnaire and training load. HI 30 minutes before each training session and RPE 30 minutes after each training session by wrestlers were individually reported to the researchers [3]. The RPE number multiplied by the training time of each session was used to calculate the training load of the s-RPE. From this variable, the other indicators of training load – AW, CW, ACWR, TM, TS [2, 3] – were calculated for the whole PS. Information about the health status (i.e., HI) of juvenile wrestlers was obtained by asking them individually before each session [17, 18]. This included: quality of sleep, feeling exhausted (fatigue), contusion, DOMS, and stress status [13, 19]. These variables were calculated on a weekly basis and afterward were used for analyses. The unit of all variables of this study is the selected arbitrary unit (AU). Intermittent Fitness Test 30–15 (IFT_30–15_) was used to calculate the VO_2max_ of the subjects. After performing the test, the formula specific to IFT_30–15_ was used to obtain the VO_2max_ [20]. For this test, test-retest reliability was performed and afterward the intra-class correlation coefficient was calculated and was 0.81.

**TABLE 1:** During monitoring in full season.

Years	2017																								2018							
W (n)	1	2	3	4	5	6	7	8	9	10	11	12	13	14	15	16	17	18	19	20	21	22	23	24	25	26	27	28	29	30	31	32
TS (n)	7	6	6	7	7	3	7	6	7	6	7	7	7	7	6	7	7	7	7	6	7	7	7	7	7	6	7	6	7	7	7	5
Months	July				August				September				October				November				December				January				February			
Periods	Early-PS											Mid-PS												End-PS								

**Note:** W, week; TS, training session; Early-PS, Early-preparation season, Mid-PS; Mid-preparation season; End-PS, End-preparation season.

### Procedures

*Anthropometrics*: Anthropometric measurements were taken once in the morning before the study [13, 21]. Standing height and body mass were measured (Seca 654, Hamburg, Germany). The accuracy of the height gauge was ± 5 mm and that of the scale was 0.1 per kg. These measurements followed the International Society for the Advancement of Kinanthropometry advanced instructions [22]. All measurements were repeated twice and their means were recorded. If the technical error measurement between the two assessments was above 3%, then the measurement was performed for the third time and the median of these three replications was finalized and recorded [13, 15].

*Monitoring internal training loads:* The internal loads were calculated by means of the s-RPE. In particular, the daily training load was quantified by means of the Borg scale re-designed by Foster [23]. To obtain the RPE score of each wrestler, the following question was regularly asked: “How do you feel about practising?” The announced number was recorded by RPE according to the Borg questionnaire (0 to 10). According to previous studies by Borg and Foster [16, 24, 25], the RPE score was obtained between 20 and 30 minutes after the end of the training sessions [26]. The score obtained by the athletes in RPE were then multiplied by the time of the session in minutes, thus providing the s-RPE measured in arbitrary units (A.U.) [27]. Wrestlers had been experienced in reporting RPE for at least 3 years.

*Calculate workload indices:* wAW is equal to the sum of the total weekly training loads [28]; the wCW is equal to the average training load in the last three weeks. Due to the use of this formula, this variable is available from the third week [29]. The wACWR was calculated using the uncoupled formula as in previous studies [13, 29, 30]. Due to the use of this formula, this variable is available from the fourth week. For example, wACWR for week 5 is equal to wAW5/0.333 × (wCW in the previous 3 weeks); wTM is equal to the average training load obtained per week divided by the SD of the training load per week. wTS is equal to wAW divided by wTM [28, 31].

*Wellbeing status monitoring:* The HI is a four-variable questionnaire (stress, fatigue, DOMS, and sleep quality) with seven points. Studies have shown that it can monitor an athlete’s wellbeing status [17, 32]. This questionnaire was used before the training sessions of athletes to record the situation in the above four variables. Number 1 in this questionnaire means good condition and number 7 means bad condition in that index for the athlete. The accumulated data weekly of these variables were analysed: wStress, wFatigue, wDOMS, wSleep, and wHI. Wrestlers had experience using this questionnaire in previous years. All questions were asked individually. The daily data register was made in Excel.

### Statistical analysis

SPSS (version 22.0; IBM, Armonk, NY) was used for computations. Descriptive statistics are presented as mean and SD. Additionally, the weekly changes and coefficients of variation are shown as percentages. Data normality and homogeneity were checked applying the Shapiro-Wilks and Levene’s tests, respectively. After that, the associations between training workload measures and wellbeing variables were evaluated using the Pearson correlation test (r). Correlation thresholds were defined as follows [33]: ≤ 0.1, trivial; > 0.1 to ≤ 0.3, small; > 0.3 to ≤ 0.5, moderate; > 0.5 to ≤ 0.7, large; > 0.7 to ≤ 0.9, very large; and > 0.9, nearly perfect. The correlations were always presented with the 95% confidence interval (CI). The alpha level was set at P ≤ 0.05. To calculate an a-priori estimation of power and sample size, the statistical software G-Power (University of Dusseldorf, Dusseldorf, Germany) was applied. The selected study design: t tests – Correlation: Point biserial model.

## RESULTS

**FIG. 1:**
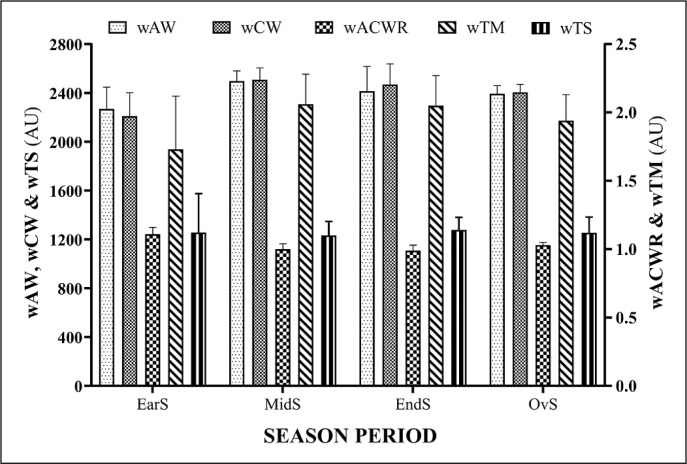
Summary of every season period for training load variables: weekly acute workload (wAW), weekly chronic workload (wCW), weekly acute/chronic workload ratio (wACWR), weekly training monotony (wTM) and weekly training strain (wTS) Arbitrary Units (AU), Early-preparation season (EarS), Mid-preparation season (MidS), End-preparation season (EndS), Overall-preparation season (OvS=Full season).

**FIG. 2:**
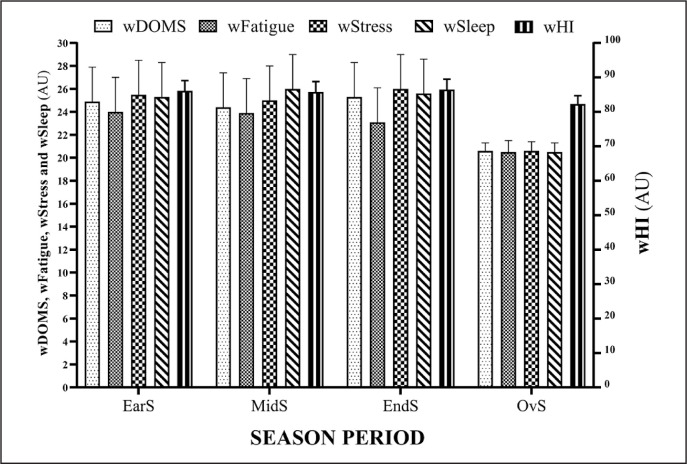
Summary of every season period for wellbeing variables: weekly sleep quality (wSleep), weekly delayed onset muscle soreness (wDOMS), weekly fatigue (wFatigue), weekly stress (wStress) and weekly Hooper indicator (wHI). Arbitrary Units (AU), Early-preparation season (EarS), Mid-preparation season (MidS), End-preparation season (EndS), Overall-preparation season (OvS=Full season).

Figures 1 and 2 show the summary of every season period for training load variables (wAW, wCW, wACWR, wTM and wTS) and wellbeing variables (wDOMS, wFatigue, wStress, wSleep and wHI), respectively.

**FIG. 3:**
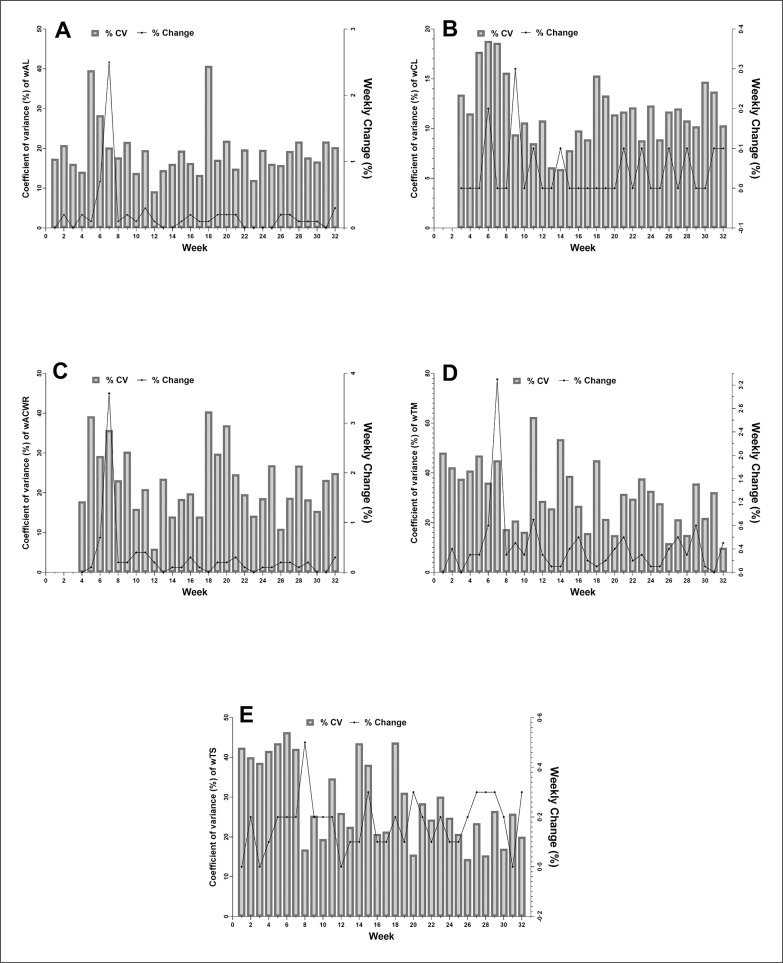
Within-week coefficient of variations (CV) and between-week variations (%) for A) acute workload (AW), B) chronic workload (CW), C) weekly acute/chronic workload ratio (wACWR), D) weekly training monotory (wTM) and E) weekly training strain (wTS) across the season.

Within-week coefficient of variation (CV) and between-week variation (%) values for wAW, wCW, wACWR, wTM and wTS during in PS periods are presented in Figure 3. The maximum (41%) and lowest (9%) CV occurred in w18 and w12, respectively (Figure 3A). The greatest between-week change happened from w6 to w7 (Figure 3A). The highest (19%) and minimum variation (6%) was observed in w6 and w14, respectively for wCW (Figure 3B). For wACWR, the maximum (40%) and minimum (6%) CV was recorded in w18 and w12, respectively (Figure 3C). The greatest between-week variations occurred from w8 to w9 for wCW and w6 to w7 in wACWR (Figures 3B and 3C). The largest increase in between-week variation was observed from w6 to w7 for wTM, and from w7 to w8 for wTS. wTM and wTS presented the greatest CV (w11-TM: 62%; w6-TS: 46%) and smallest (w32-TM: 10%; w26-TS: 14%) (Figures 3D and 3E).

**FIG. 4:**
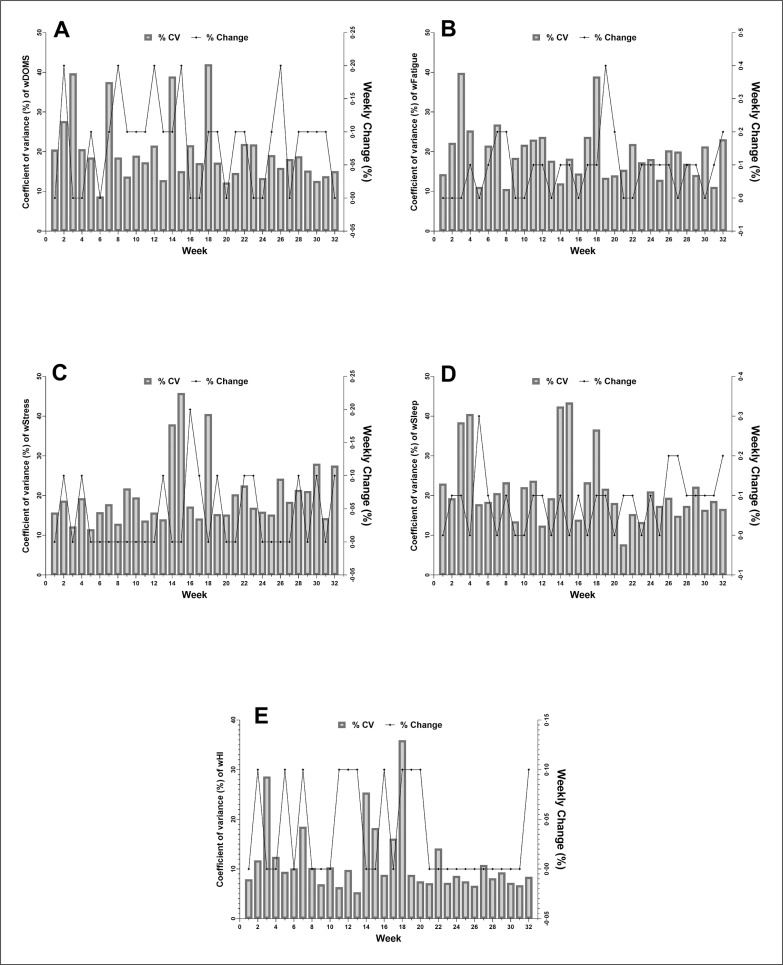
Within-week coefficient of variations (CV) and between-week variations (%) for A) weekly delayed onset muscle soreness (wDOMS), B) weekly fatigue (wFatigue), C) weekly stress (wStress), D) weekly sleep quality (wSleep), and E) weekly Hooper indicator (wHI) across the season.

Figure 4 shows the within-week CV and between-week variations (%) for wellbeing categories across the PS. The CV in wDOMS was observed to be highest in w18 (42%) and lowest in w26 (9%) (Figure 4A). The greatest between-week change happened from w25 to w26 (Figure 3A). The highest CV was observed in w3 (40%) and the minimum variation (11%) in w8 for wFatigue (Figure 3B). wStress had the CV maximum in w15 (46%) and minimum in w5 (12%); see Figure 4C. The greatest between-week variation occurred from w18 to w19 for wFatigue, and w15 to w16 for wStress (Figures 4B and 4C). The percentage change in between-week variation showed the largest increase from w4 to w5 for wSleep, and w18 to w19 for wHI. Ultimately, wSleep and wHI presented the maximum CV (w15-sleep: 43%; w18-HI: 35%) and insignificant (w21-sleep: 8%; w13-HI: 5%) (Figures 4D and 4E).

**FIG. 5:**
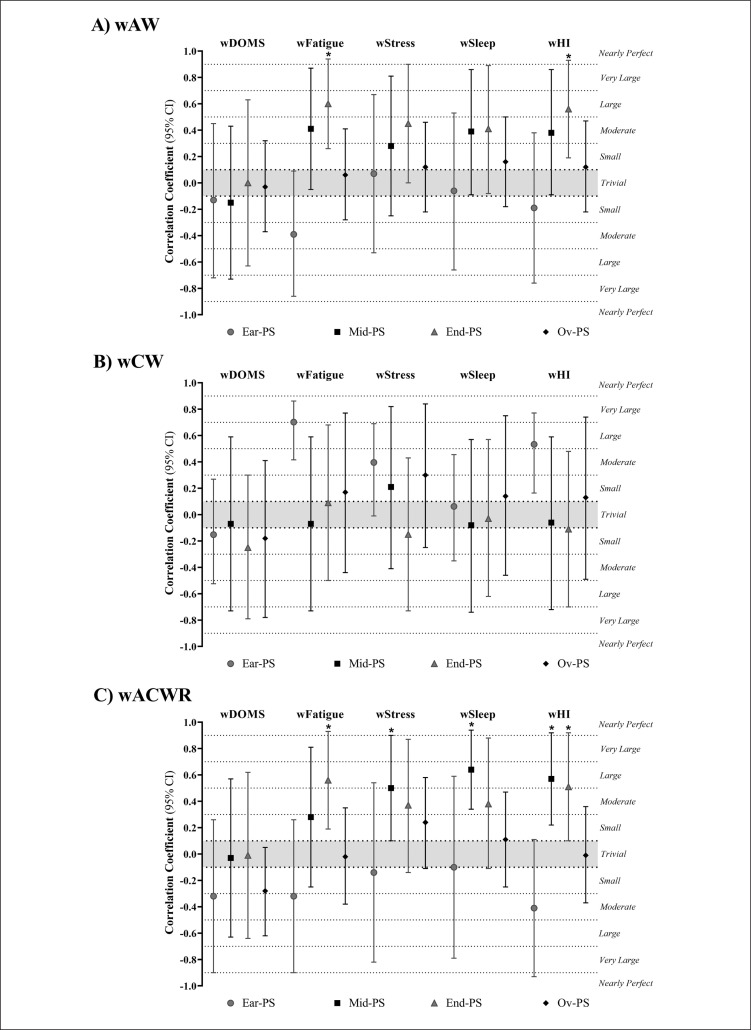
Correlation coefficients (95% CI) of A) weekly acute workload (wAW), B) weekly chronic workload (wCW) and C) weekly acute/ chronic workload ratio (wACWR) and wellbeing categories during early-preparation season (Ear-PS), mid-preparation season (Mid-PS), end-preparation season (End-PS) and Overall-preparation season (Ov-PS). wSleep, weekly sleep; wDOMS, weekly delayed onset muscle soreness; wFatigue, weekly fatigue; wStress, weekly stress; wHI, weekly Hooper indicator. * Correlation coefficient is significant at p-value ≤ 0.05.

Associations of wAW, wCW, and wACWR with wellbeing variables for the early-, mid- and end-PS periods and the full PS are presented in Figure 5. Overall, wAW had significant very large correlations with wFatigue (r = 0.77; 95% CI: 0.26 to 0.94; *P* = 0.01) and wHI (r = 0.73; 95% CI: 0.19 to 0.93; *P* = 0.02) during end PS (Figure 5A). wACWR had significant very large and large correlations with wFatigue (r = 0.73; 95% CI: 0.19 to 0.93; *P* = 0.02) and wHI (r = 0.69; 95% CI: 0.09 to 0.92; *P* = 0.03) during end PS. Workload parameters with meaningful correlations were wStress (r = 0.66; 95% CI: 0.10 to 0.90; *P* = 0.03) with a large correlation, wSleep (r = 0.78; 95% CI: 0.35 to 0.94; *P* = 0.004) with a very large correlation, and wHI (r = 0.72; 95% CI: 0.22 to 0.92; *P* = 0.01) with a very large correlation during mid PS (Figure 5C). There were no significant correlations between wCW and wellbeing variables during full-PS periods (Figure 5B).

**FIG. 6:**
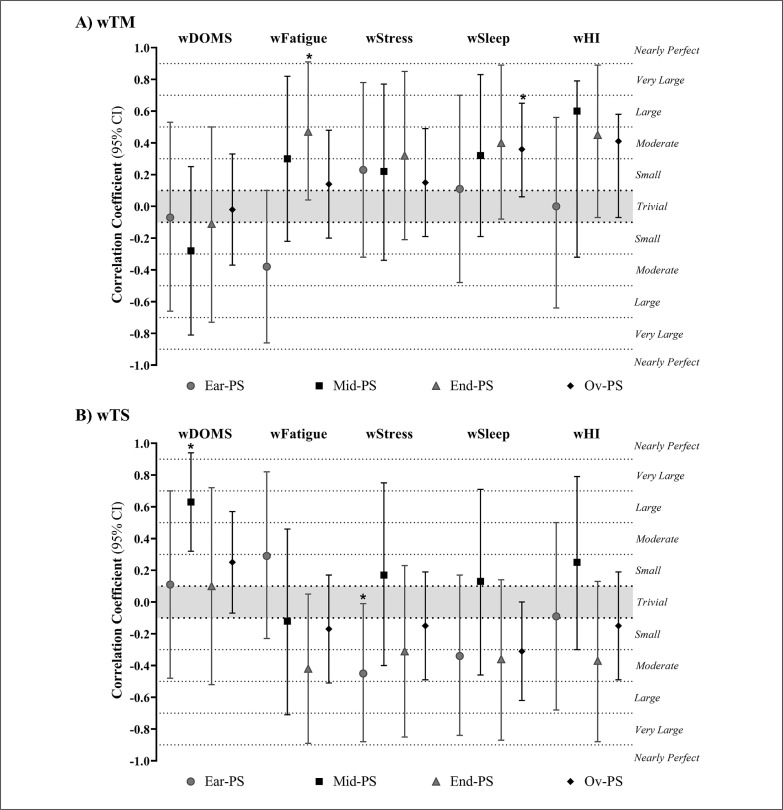
Correlation coefficients (95% CI) of A) wTM (weekly training monotony) and B) wTS (weekly training strain) and wellbeing categories early-preparation season (Ear-PS), mid-preparation season (Mid-PS), end-preparation season (End-PS) and Overall-preparation season (Ov-PS). wSleep, weekly sleep; wDOMS, weekly delayed onset muscle soreness; wFatigue, weekly fatigue; wStress, weekly stress; wHI, weekly Hooper indicator. * Correlation coefficient is significant at p-value ≤ 0.05.

The relationship between wTM, wTS and wellbeing status is shown in Figure 6. wTM showed a large correlation with wFatigue during end PS (r = 0.65; 95% CI: 0.04 to 0.91; *P* = 0.04) and a moderate correlation with wSleep in the overall PS (r = 0.40; 95% CI: 0.06 to 0.66; *P* = 0.03). wTS presented a large correlation with wStress during early PS (r = 0.61; 95% CI: -0.88 to -0.01; *P* = 0.048). wTS showed a very large correlation with wDOMS during mid PS (r = 0.77; 95% CI: 0.32 to 0.94; *P* = 0.005) (Figure 6B).

## DISCUSSION

The objectives of this study were: (1) to analyse the relationships among wAW, wCW, wACWR, wTM, wTS, DOMS, fatigue, stress, sleep quality, and HI; (2) to analyse the relationships in different periods of the early, mid and end PS with the full PS.

In our study higher variation of wAW, fatigue, stress and HI was observed in the end PS. The most significant variation in wCW and fatigue was observed in the early PS, but the most significant reduction was observed at the mid PS. The largest increase and reduction in wACWR were observed in the early PS, where there was also the largest reduction in wTM, wTS, wDOMS, wStress and wHI. It is in concordance with previous studies; Clemente et at. also found the highest reduction of wDOMS, wStress and wHI during the early season. It seems that during the beginning of the season there are fewer decisive matches and this could improve the wellbeing [29].

wTM, wTS, wSleep and wDOMS showed the greatest variation in mid PS. The highest value of between-week variation for wTS was w8 to w9, and the lowest reduction for wACWR was between w9 and w10. The highest within-week variation was found for wACWR in w18, and the lowest for wCW in w19. The results of this study are somewhat different from previous studies in young soccer players [13]. Nobari et al. reported that the highest values of variation of wCW and wTS were observed in the mid season, while the lowest values were found in the early season. They also observed that the highest values of wFatigue, wDOMS, and wStress corresponded to the end season, and the lowest values of wSleep and wStress were in the early season, while the lowest values of wFatigue and wDOMS were observed in the mid season [13]. The discrepancies could be related to the difference between team and individual sport.

We found small differences in correlations between wTM, wTS and wellbeing. In line with this, Sawczuk et al. did not find a relationship between daily wellbeing and training loads in young athletes, but wellbeing was associated with low sleep duration while perceived recovery status did not show any association with sleep duration [34]. Hartwig et al. (2009) also did not find significant correlations between training load and well-being status in young rugby players [35]. It could be explained by two reasons. First, our research sample consists of young athletes. It is possible that this situation, which affects the results obtained, is caused by the difference in the relative stressor intensity of adult and young athletes. Young athletes have a unique environment for social, educational, and maturing conditions to navigate [36]. This environmental situation, which positively affects the well-being of young athletes, could be more important for them than their training. The second possible reason is that the participants in other studies are generally considered to be team athletes. Different physiological and kinematic needs of team sports athletes may have affected the results.

In our study the wTM was largely correlated with wFatigue during end PS and moderately correlated with wSleep in the overall PS. Our results agree with the study of Clemente et al. They found a similar correlation between wTM and wDOMS (r = 0.80), wSleep (r = 0.72), and wFatigue (r = 0.82) in volleyball players [37].

There was a correlation of wTS with wStress during early PS and with wDOMS during mid PS. Previous studies also found that stress was sensitive to training load [38]. Lathlean and colleagues [39] confirmed that larger variation in load with lowered monotony was associated with higher DOMS. Additionally, Bok et al. reported a stronger relation between training load and stress state [26]. In another study, Lupo et al. [12] found that a large variation of training load can be followed in the individual week-to-week fluctuations, whereby the single fluctuations can be significantly different from the overall mean of the group. In another study, Collette et al., Lupo et al. [12] found that a large variation of training load can be followed in the individual week-to-week fluctuations, whereby the single fluctuations can be significantly different from the overall mean of the group.

## CONCLUSIONS

The results of this study provide new insights for coaches and practitioners, not only for those in Turkey, about the perceived load level and the variations of wellbeing over a season at elite youth level. Stronger associations between workload and wellbeing variables of young soccer players in the early season were also revealed. Thus, a greater consideration should be given to better control the training process and maximize the development of young players. Thereby, wellness variables may be considered as a useful tool to provide determinant psychological states of players’ information to the coaches and thus possibly identify important variations in training responses. However, in future work, we intend to assess whether the results of our study are generalizable to all teams or whether the loading effort, during a season, is perceived differently depending on individual characteristics and training of elite youth. We also intend to increase the number of features recorded in each training session or match and to perceive as correctly as possible the level of load and the variations of well-being of the athletes.
